# Mining a stroke knowledge graph from literature

**DOI:** 10.1186/s12859-021-04292-4

**Published:** 2021-07-29

**Authors:** Xi Yang, Chengkun Wu, Goran Nenadic, Wei Wang, Kai Lu

**Affiliations:** 1 College of Computer, National University of Defence Technology, Changsha, 410073 China; 2 State Key Laboratory of High-Performance Computing, National University of Defence Technology, Changsha, 410073 China; 3 Department of Computer Science, University of Manchester, Manchester, M13 9PL UK

**Keywords:** Stroke, Knowledge graph, Biomedical text mining, Traditional Chinese Medicine

## Abstract

**Background:**

Stroke has an acute onset and a high mortality rate, making it one of the most fatal diseases worldwide. Its underlying biology and treatments have been widely studied both in the “Western” biomedicine and the Traditional Chinese Medicine (TCM). However, these two approaches are often studied and reported in insolation, both in the literature and associated databases.

**Results:**

To aid research in finding effective prevention methods and treatments, we integrated knowledge from the literature and a number of databases (e.g. CID, TCMID, ETCM). We employed a suite of biomedical text mining (i.e. named-entity) approaches to identify mentions of genes, diseases, drugs, chemicals, symptoms, Chinese herbs and patent medicines, etc. in a large set of stroke papers from both biomedical and TCM domains. Then, using a combination of a rule-based approach with a pre-trained BioBERT model, we extracted and classified links and relationships among stroke-related entities as expressed in the literature. We construct StrokeKG, a knowledge graph includes almost 46 k nodes of nine types, and 157 k links of 30 types, connecting diseases, genes, symptoms, drugs, pathways, herbs, chemical, ingredients and patent medicine.

**Conclusions:**

Our Stroke-KG can provide practical and reliable stroke-related knowledge to help with stroke-related research like exploring new directions for stroke research and ideas for drug repurposing and discovery. We make StrokeKG freely available at http://114.115.208.144:7474/browser/ (Please click "Connect" directly) and the source structured data for stroke at https://github.com/yangxi1016/Stroke

**Supplementary Information:**

The online version contains supplementary material available at 10.1186/s12859-021-04292-4.

## Background

Stroke, also known as cerebrovascular accident (CVA), is a group of diseases with three major types (hemorrhagic stroke, ischemic stroke, and TIA transient ischemic attack) and with cerebral infarction being the most common phenotype [1]. In the past decades, stroke treatment and prevention have seen significant advances in particular in declining stroke mortality [2]. Western therapeutic such as drug injection and endovascular therapy [3], as well as traditional Chinese treatment such as herbal medicine and acupuncture [4], have made tremendous efforts for preventing stroke and recovery after stroke. However, stroke is still one of the most critical fatal diseases worldwide (the second leading cause of death) [5] because of acute onset, with the enormous economic burden of recovery for those who survive. So, there is a need to investigate potential pathogenic genes, risk factors further, and aura symptoms of stroke to find efficient preventative and therapeutic approaches.

There are some existing structured knowledge sources focused on stroke [6–8]. Still, a large amount of stroke-related information is available in scientific articles. For example, a recent search for ‘*stroke*’ in PubMed resulted in over 327 K papers. In this study, we aim to develop a stroke-related knowledge base by combining information extracted from these scientific papers and existing knowledge bases. The large volume of texts requires automated and computational methods to extract useful information from these unstructured data to build structured databases.

Knowledge graphs (KGs) [9] are widely known as knowledge domain visualization or knowledge domain mapping graphs in the library and information industry [10]. They are often represented as a series of different graphs with the relationships between development processes and the structure of knowledge. Visualization technology is used to describe, analyze, construct, and display knowledge and inter-relationships [11]. Such representation methods can promote the understanding of relations between biomedical entities, which is vital for scientific researchers to refine their research scope and improve personalized medicine. It is also possible to discover new knowledge (e.g., new drugs [12] and effective prevention/treatment methods [13, 14]). However, it is a laborious and time-consuming process to construct a KG manually. Therefore, automated approaches to assist an automated/semi-automated construction of knowledge graphs in specific domains have been used [15, 16].

In this paper we introduce a stroke-related knowledge graph (StrokeKG) by combining information extracted from these scientific papers and existing knowledge bases. In addition to biomedical entities, we also add entities from Traditional Chinese medicine (TCM) [17], which pays close attention to the medical characteristics of the entire system of the human body, which makes it a promising candidate for the treatment of stroke [18]. We use a suite of tools to extract genes, diseases, drugs, symptoms, Chinese herbal medicine, and other entities and link them using relationship extraction methods. As a result, StrokeKG includes 46,983 nodes of 9 types, and 157,302 relationships of 30 types, connecting diseases, genes, symptoms, drugs, pathways, Chinese Patent Medicines (CPMs), Herbs, Chemical, ingredients. Besides, we marked 265 CPM entities and 404 CPM-Disease relationships through verification and manual annotation of existing databases to provide practical and accurate stroke-related knowledge. The graph can be used to facilitate our understanding of this complex disease, for example, by exploring precursor symptoms and sequelae of stroke, therapeutic drugs, and the pathway for treating related diseases.

## Related work

In the field of biomedicine, knowledge bases (KBs) such as Gene ontology [19], disease ontology [20], reference terms for national drug archives [21], and basic models of anatomy [22] have been prominent examples of efforts to provide structured knowledge systematically. Some of these KBs, e.g. OpenKG [23], BenevolentAI [24], and KnowLife [14] have made significant contributions to the development of the biomedical field, including recent drug repositioning for COVID-19 [25], SemaTyP [26], and protein-drug target KG [15] have been used. Despite many efforts to provide more structured data, vast amounts of relevant knowledge are still hidden in the biomedical literature [27]. There are three main limitations to previous work on KB construction [9]. First, most biomedical KBs are manually constructed and curated, which defer them from keeping up with the pace of novel discoveries. Second, potentially useful text sources such as health portals, online communities, or other sources of information are often ignored. Finally, most previous works focused on one molecular level or chemical genomics, such as protein–protein interactions [28], gene-drug relationships [29], or just highly specific topics such as drug effects.

Natural language processing tools are indispensable to extract useful information from biomedical literature [30]. We need to start with the named entity recognition process and then relationship extraction. Biomedical Named Entity Recognition (NER) [31] aims to identify specific biomedical concepts in the text. NER consists of two steps: (1) classifying specific substrings obtained from the text to determine whether it is the name of a specific type of entity; (2) selecting a standard name or a unique identifier for one kind of entity [32]. There are already many NER tools available for different types of biomedical entities, such as genes/proteins [33], diseases [34, 35], species [36], mutations [37], chemicals [38, 39] and biological pathways [40]. Still, many essential concept types such as RNAs, phenotypes, Chinese Patent Medicines (CPMs), and herbal medicines do not have corresponding NER tools.

The task of Relation Extraction (RE) is has been in the focus of research in recent years. Due to the inherent complexity of the biomedical text, most relation extraction systems work at the sentence-based level. Common relationships include protein–protein interactions [28], drug–drug interactions [41], gene regulatory events [42], associations between mutations and diseases [43]. Early relationships used a co-occurrence approach [44], while pattern-based systems [45] rely on a set of manually or automatically collected patterns to extract relations and classify relation types between entities. Rule-based methods [46, 47] use a set of processes or some heuristic algorithms to manually define or build a set of rules based on domain experts and automatically generated from the training data. It adds multiple constraints to scope specific relationships: for example, BioNLP'09 [48] focused on nine common molecular events. More recently, with the improvement of the accuracy and expanded availability of curated corpora, deep learning models are widely used in the field of natural language processing. Convolutional Neural Network (CNN) [49], Recurrent Neural Network (RNN) [50], Long Short Term Memory Network (LSTM) [41], Capsule Network, CapsNet [51], Graph Neural Networks [52, 53], and BERT [54] are prevalent models employed in relation extraction, making great contribution to biomedical text mining.

For the field of traditional Chinese medicine, Manually organized TCM database, TCMID [55], TCM-MESH [56], and *Chinese medicine network pharmacology* ETCM [57, 58], TCMSP brings convenience to the research of Chinese medicine. However, to the best of our knowledge, there is no text mining tool specifically for Traditional Chinese medicine, and there is also the non-disclosure or incomplete knowledge in stroke-related knowledge [6–8]. Therefore, in this research, we will enrich the application of text mining in the construction of Chinese medicine knowledge, and based on this and the-start-of-art, construct a stroke-related knowledge graph.

## Methods

In this work, we designed a computational workflow to mine the stroke-related and TCM-related literature for the identification of biomedical entities and the relations between them. We split stroke-related abstracts into 463,225 sentences, the analysis pipeline tags the mentions of the following entities: drugs, chemicals, genes, pathways, and diseases, as well as traditional Chinese treatments like Herbs, Chinese Patent Medicines (CPMs), and ingredients. To increase the data set of Chinese medicine on stroke-related disease, we then split TCM-related abstracts into sentences for extract disease, CPM and herbs. We then use several approaches to relations between entities. After verifying and cleaning of the results, we use NEO4J to construct StrokeKG.

**Fig. 1:**
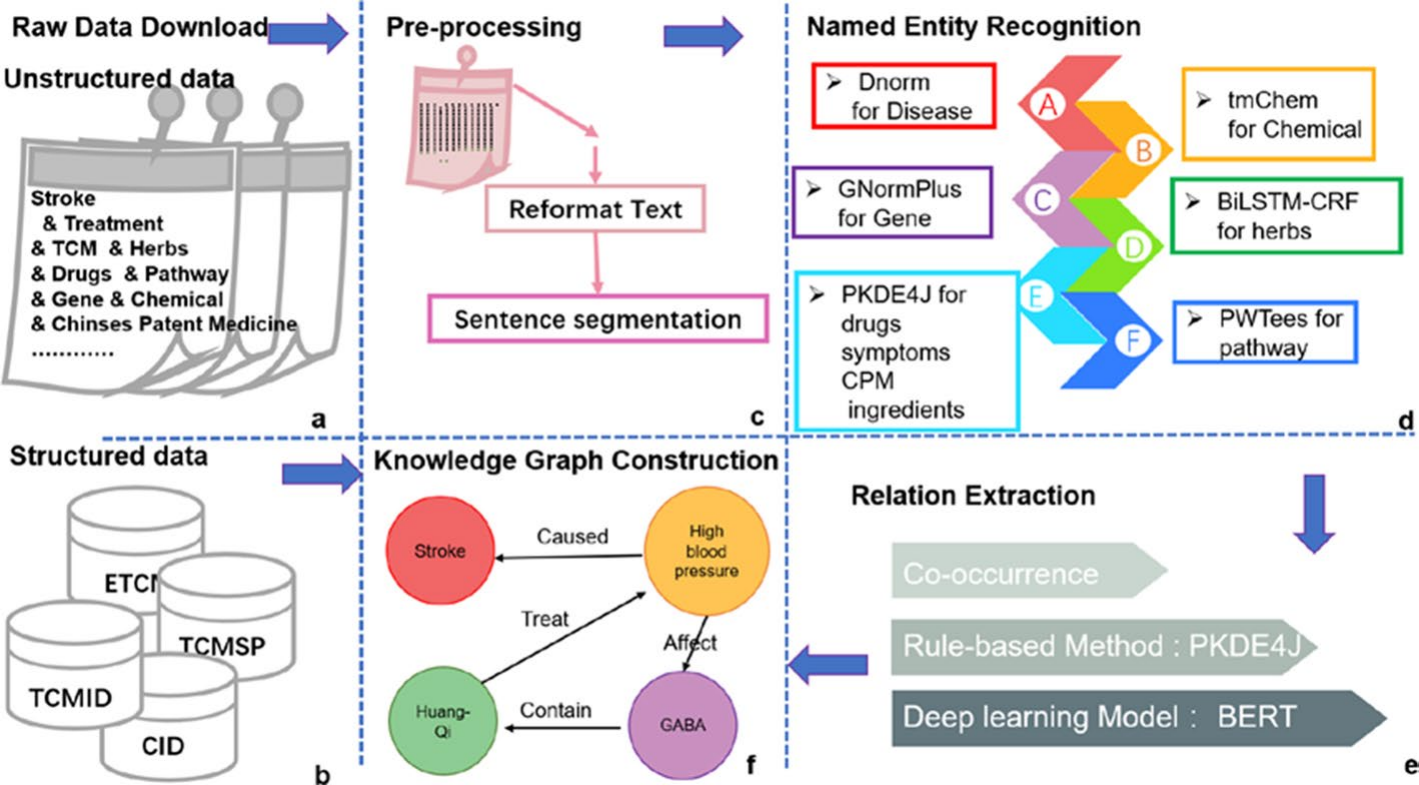
The workflow for constructing StrokeKG. **a** Unstructured data search, **b** structured data download, **c** pre-processing, **d** Named Entity Recognition, **e** Relation Extraction, **f** Knowledge Graph construction

The steps of our workflow are explained below (Fig. 1).

### Data source

A search for “Stroke AND treatment OR gene OR Herbs OR TCM” in PubMed resulted in 45,080 stroke-related and “Traditional Chinese Medicine” 72,410 TCM related abstracts, which we used as a dataset to extract information from. In addition, manually created databases and annotated corpora, drug-disease relation database: CDR [59], CTD [60], gene-disease relation corpus: EU-ADR [42], and TCMID ETCM [57], TCMSP [58] are also the main source of our knowledge graph data. Table 1 details the data source of our research.

**Table 1:** Data resource of our StrokeKG

Data source	Types	Number of entities	Number of relations	Number of documents
PubMed	English abstracts	–	–	52,175 abstracts
CTD [60]	Western medicine databases	Chemical (19, stroke-related)	19 (stroke-related)	19 (stroke-related) 470(total)
CDR [59]	Western medicine corpus	Chemical (14, stroke-related) Chemical (1279, total) Disease (1188, total)	15 (stroke-related) 3116 [42] (total)	10 (stroke-related) 1500 (total)
TCMID	TCM databases	CPM (681)	–(therapeutic)681	–
ETCM [57]	TCM databases	Gene(10), Herbs (498), CPM (3419)	–	–
TCMSP [58]	TCM databases	Gene (63), Herbs (114)	–	–
DDI corpus [61]	Western medicine corpus	–	Drug-drug:14,281	
EU-ADR [42] corpus	Western medicine corpus	–	Gene–disease:355	100 Medline abstracts
Plant-disease corpus [62]	TCM databases	Disease:100 Plant:1102	Plant-disease: Therapeutic (Treatment):708 Induce(Cause):486	180 abstracts

Stroke MESHID:D020521, cerebrovascular ischemia/Ischemic stroke/ brain ischemia D007511, cerebral ischemia D002545, transient ischemic attack(TIA): D002546, haemorrhagic stroke/subarachnoid haemorrhage MESH:D013345, cerebrovascular accident(CVA) D002544, Subarachnoid hemorrhageMESH:D01334 D002543

### Pre-processing

We re-formatted the PubMed abstracts into the PubTator [63] format to match the data for NER tools and then split sentences by NLTK [64].

### Named entity recognition

We extract mentions of nine named-entity types (diseases, drugs, genes, symptoms, pathways, Chinese Patent Medicines (CPMs), Herbs, Chemicals, Ingredients). We use state-of-the-art NER methods, including DNorm [34] to extract and normalize disease words, tmChem [38] as a chemical named entity identifier, GNormPlus [33] to handle both gene mentions and identifier detection, and pathways through PWTEES [40].

We used a pre-trained BiLSTM-CRF [65] model with the Plant-disease corpus [62] to build a NER classifier to identify Herbs. The lack of annotated corpora poses a considerable challenge to using deep learning methods to build other NERs needed for our study. We have therefore developed dictionary- and rule-based methods for other entity types. A rule-based method PKDE4J [46] was used to modify the Stanford CoreNLP pipeline to extract entities based on drug dictionaries. Which we have collected Symptoms and ingredients are recognized by collecting terms from download the CPM database [55] and the ingredient database[56], construct a symptoms dictionary, and which are then inserted the dictionary into the PKDE4J model applied a dictionary-based method for NER.

To eliminate the occurrence of an entity by accident, we determine the threshold based on the number of occurrences of the entity. When the number of occurrences of the entity is smaller than 3, We will manually determine whether the entity is related to stroke.

### Relation extraction

We focus on eleven relationship types as specified in Table 2. These have been taken from the existing databases and from existing corpora (see below).

**Table 2:** Keyword classification rules and corpus for drugs/TCM/Herbs/chemicals and diseases

Entity pairs	Relation types	Associated Keywords	Corpus
CPM-Disease/Symptoms Herbs-Disease/Symptoms Drug-Disease/Symptoms Chemical-Disease/Symptoms	Treatment	Therapy, treating, cure, remedy Inhibit…	Plant-disease Corpus
	Cause	Induce, cause, side effect, influence, dynamic…	
	other	No special key word…	
CPM-Herbs CPM-Chemicals Herbs-Chemicals CPM-Drugs Herbs-Drugs	advise	Avoid, should not be	Drug–Drug Interaction Corpus
	int	Interaction, and, between	
	effect	Enhance, against, demonstrated	
	mechanism	Metabolize, decrease, increase	
	negative	no	
	Entity-Origin	Include, contain,	
Gene-Disease	Positive association (PA)	Effect, induce, target	EU-ADR
	Negative association (NA)	Indifference, no	

**Fig. 2:**
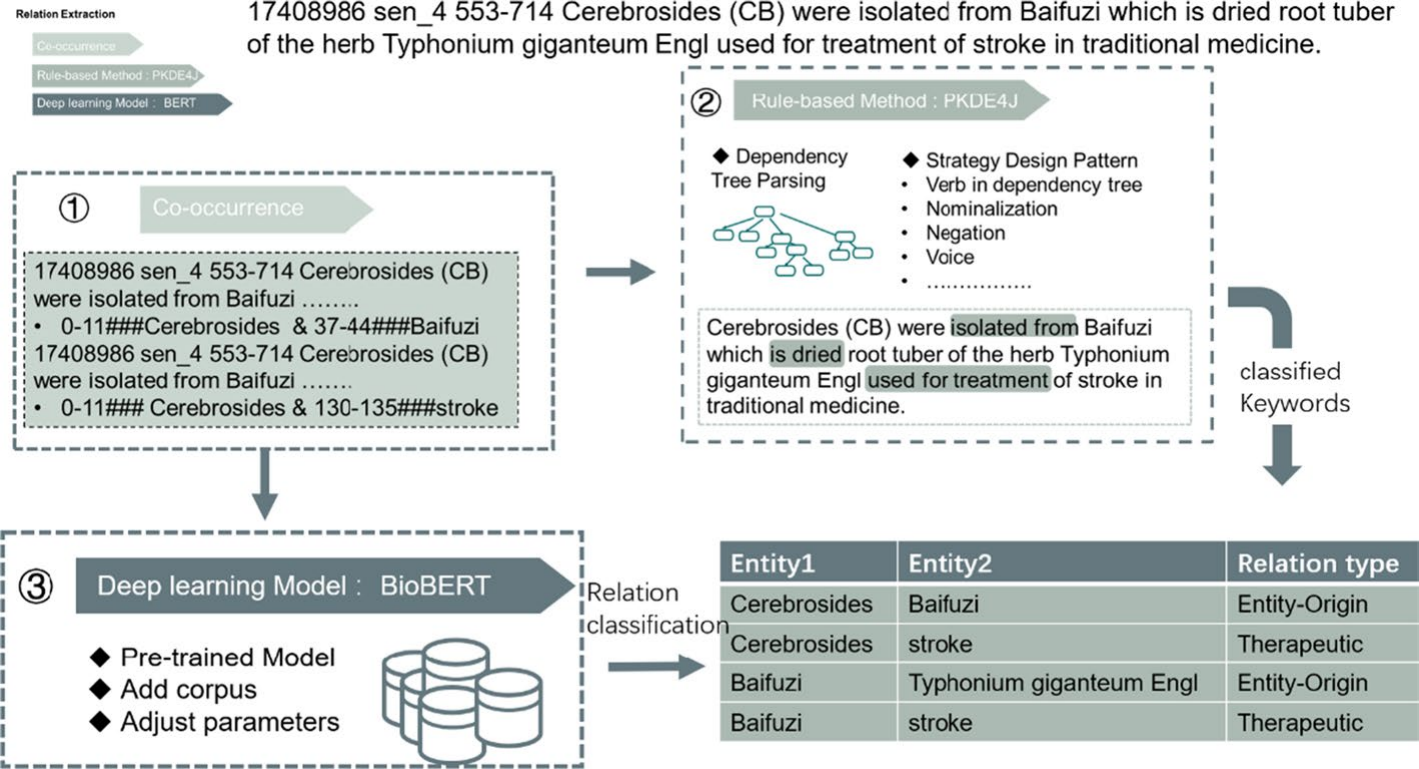
Pipeline and details about relation extraction

The relation extraction process is shown in Fig. 2. We first use a simple co-occurrence method. When two entities appear in the same sentence, we consider that there is a particular relationship between them. Secondly, a rule-based method has been used to extract ‘evidence’ for the relationship between two entities. Finally, we developed a machine-learning model to further classify relation types according to existing databases or corpora.

- Co-occurrence extraction
  We use NLTK [64] to segment each sentence and match the position of each entity in the sentence according to the entity positions determined by multiple NER model (see Fig. 2①).
- Rule-based approach
  We used PKDE4J [46] to create a dependency tree containing syntactic and grammatical structures. We rely on standard features and structures in sentences that may represent relationships and extract the keywords that may express the relationship between two entities identified via co-occurrence (Fig. 2②). We then designed a set of matching rules to classify these keywords to elven relationship types (e.g., positive association; therapeutic; induce; etc.) between specific pairs of entities (e.g., Gene-Disease; Herb-Chemical) as specified in the existing biomedical databases (e.g., TCMID [55], CTD database [60]) (as shown in Table 1).
- Extracting relation by Bio-BERT

We chose Bio-Bert [54] as a pre-trained model, which shares potential latent features with our data as it was re-trained on biomedical corpora. According to the parameter configuration of BioBERT, we use the gold standard data sets [42, 60, 61] as the training sets and our **Co-occurrence** results as the test set and select the result of the 20th epoch as the final result of our relation extraction. The corpora for relationship extraction we use can be seen in Table 2.

The co-occurrence method proves that two entities appear in the same sentence, indicating that there is a possible relationship between the entities. Rule-based methods can classify entity relationships well if keywords are extracted. When the keywords cannot be extracted, we use BioBERT's classification results, which can classify all relationships, but it much depends on the richness of the corpus and the accuracy of the model.

Because entity pairs may appear in different sentences, the classification results may differ. To find all relations between the pair of two entities, we calculated the confidence for the pair related by a particular relation, overall the sentences in which that pair of entities co-occur. We select only those relationships with confidence more considerably than the threshold to eliminate the noisy relationships that happen by accident. Afterward, we analyze the final relationship results for the entity.

### Manual annotations for TCM corpus

#### Entity annotation

To verify the effectiveness of our Chinese herbal medicine related entity mining tool. This work mainly focuses on the annotation of herbs and Chinese Patent medicine in 450 TCM-related abstracts. We regard mentions mined by the tool as pre-annotation of entities. Therefore, according to the vocabulary provided by TCMID [55] and ETCM [57], we only need to modify the incorrect annotations and add annotations to the undetected entities, instead of annotating entities from scratch.

The definition of the target entities we are concerned with is as follows:

*Chinese Patent medicine: including* clinical prescription, TCM formulas and CPM.

#### Relation annotation

In relation annotation task, we only considered two relations between entities. For each relationship, we classified the type of relationship based on the two annotation guidelines. Once two target entities appear in the same sentence, we label the relationship between them.

*Chinese patent medicine-disease* this indicates the drug will treat the disease or induce the disease. According to Plant-disease corpus, the relationships are divided into 3 categories: treatment, cause and others.

### Evaluation of text-mined results

The evaluation of NER and RE was to compare the extracted results with the existing databases or manually annotated corpus.

For TCM-related NER tools, we compare whether the results we extracted overlap with the existing database. Secondly, for CPM entities, we will compare the results by dictionary-based tool and the results we manually annotated.

For relation extraction results, also check the overlap of the entity pair we extracted with the existing database, and then calculate the correct rate (CR) of relationship classification in the overlap section.

$$\text{Correct rate}=\frac{\text{correct classification relationships}}{\text{Overlapped relationships}}$$

### Knowledge graph construction

The construction of a knowledge graph is a compelling visual representation of entities and relationships. These are embedded in the knowledge graph to carry information about entities and relationships and are widely used in learning tasks to accelerate the completion and recommendation of the knowledge graph. By mapping the stroke-related entities from our results and existing data source (TCMID [55], CDR [66], CTD [60], TCMSP [58] and ETCM [57]) in a common ID space, we can combine these triplets into one single dataset to construct a comprehensive stroke-related repurposing knowledge graph.

## Results

### Results statistics

The results mainly include the entities and relations we mined. The statistical results and specific results of drugs, chemicals, symptoms, pathways, etc. are shown in Table 3 and https://github.com/yangxi1016/Stroke.

**Table 3:** Models and results of named entity recognition

Entity type	Entity_type/(> 3)	Entity_mentions
Disease	3250/2733	220,144
Symptoms	728/485	350,833
Chemical	7062/2246	166,701
Drugs	2156/119	102,072
Chinese Patent medicine	242/manual(265)	914
Herbs	2402/2138	7386
Ingredients	270/170	29,301
Gene	5953/3978(	180,280
Pathway	18,842	105,337

Relation extraction results statistics are in Table 4.

**Table 4:** Number of relation results by using co-occurrence and PKDE4J

	Entity1	Entity2	Relation pairs (unique pairs)	Co-occurrence	PKDE4J Keywords
1	CPM	Herbs	190	478	120
2	CPM	Ingredients	29	5	2
3	CPM	Drugs	19	30	9
4	CPM	Chemical	220	498	326
5	CPM	Symptoms	138	600	371
6	CPM	Disease	404/704(CPM-Dis++)	1031/7771	637/5439
7	CPM	Pathway	94	22	11
8	CPM	Gene	194/982(CPM-Gene++)	118	53
9	Herbs	Ingredients	515	1185	311
10	Herbs	Drugs	1545	2873	1034
11	Herbs	Chemical	2382	3926	894
12	Herbs	Symptoms	1222	4927	1974
13	Herbs	Disease	2012/12115((Herb-DIS++)	7872	2847
14	Herbs	Pathway	1303	2221	841
14	Herbs	Gene	1686	17,829	12,938
15	Ingredients	Drugs	6775	9485	1337
16	Ingredients	Chemical	3718	14,623	1965
17	Ingredients	Symptoms	1339	5513	2064
18	Ingredients	Disease	341	5224	2008
19	Ingredients	Pathway	1932	2652	870
20	Drugs	Chemical	225	844	564
21	Drugs	Symptoms	9212	27,593	12,888
22	Drugs	Disease	14,756	59,924	41,095
23	Drugs	Pathway	20,053	23,848	7836
	Drugs	Gene	22,873		
24	Chemical	Symptoms	4427	6752	2335
25	Chemical	Disease	8835	21,858	10,062
26	Chemical	Pathway	33,614	58,426	17,584
27	Symptoms	Disease	26,756	101,842	24,683
28	Symptoms	Pathway	23,536	53,599	37,897
29	Disease	GENE	10,084	84,003	16,668

### Evaluation

#### Evaluation for NER

Compared with our manually labeled CPM results, the recall, precision and F1-score of the rule-based CPM NER are shown in Table 5.

**Table 5:** F1-score of the rule-based CPM NER tool

Models	Recall	Precision	F1-score
CPM NER	86.04	94.21	90.06

The reason for the low recall mainly because of the lack of abbreviation (CY-Tang: Chungsim-Yeunja-Tang) and the different spelling of TCM caused by different pronunciation. (For example, Hwangryun-Hae-Dok-tang and Huanglian-Jie-Du-Tang).

#### Compare with existing database

**Fig. 3:**
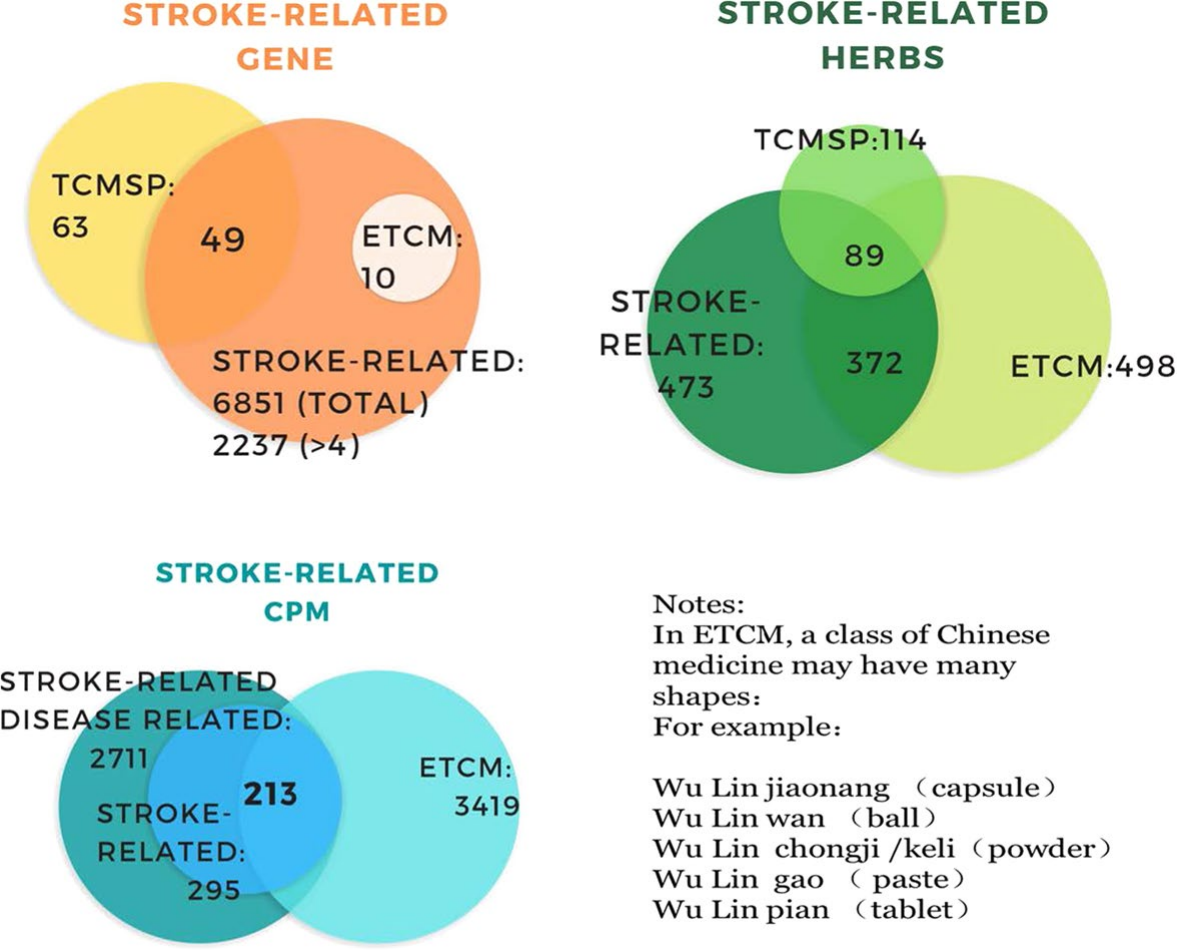
The comparison of NER results with ETCM [57] and TCMSP [58]

To assess how validity the literature-derived knowledge represented data, we compared the results to two Chinese Medicine Pharmacology Knowledge Base: ETCM and TCMSP to those obtained in StrokeKG. Including stroke-related CPM, herbs, and genes. Figure 3 shows the result of comparison with ETCM [57] and TCMSP [58].

Compared with the existing database, our name recognition results partially overlap with the existing database, which indicates that our entity recognition results are effective. More importantly, we have unearthed many stroke-related entities that do not exist in the database. Which provides a new direction for future research.

#### Evaluation for RE

Compared with our manually labeled CPM-Disease relations, the recall, precision and F1-score of the CPM-Disease RE are shown in Table 6.

**Table 6:** F1-score of the CPM-Disease RE tool

Models	Recall	Precision	F1-score
PKDE4J + BioBERT	88.92%	80.06%	84.26%

On some relationship pairs, the model cannot judge whether it is a Treatment or a Cause, and is classified as Other, which is most of the reason for the error.

**Fig. 4:**
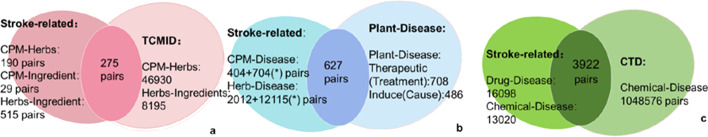
The part of our mining results that is a duplicate (comparable) with the existing database. **a** The relation between CPM-Herbs, CPM-Ingredients, and Herbs-Ingredients (Comparative database: TCMID). **b** The relation between CPM-Disease, Ingredients-Disease, and Herbs—Disease (Comparative database: Plant-disease). **c** The relation between Drugs-Disease and Chemical-Disease(Comparative database: CTD: Chemical-Disease)

As shown in Fig. 4a and Table 7, our mining results include 190 pairs of CPM-Herbs, 4 pairs of CPM-Ingredients, and 515 pairs of Herbs-Ingredients, compared with the existing TCMID (only CPM components and Herbs component table) database, there are 275 pairs of relationships that overlap and the correct rate of the relationship classification results is 91.42%. Secondly, our mining results include 404 pairs of CPM-Disease (with 704 CPM for stroke-related disease) compared with TCMID (only comparing whether herbal medicine has a therapeutic effect on the disease). The rate is 84.37%. The correct rates of the relation between genes-diseases and drugs-diseases are 90.47% and 88.86%, respectively.

**Table 7:** The correct rate of our RE results

Relation	Comparative database	number of relation overlapped	Number of correct classification	Correct rate
CPM-Herbs, CPM-Ingredients, and Herbs-Ingredients	TCMID, TCM-Mesh	275	269	97.81%
CPM-Disease, Ingredients-Disease, and Herbs–Disease	Plant-Disease	687	621	90.39%
Gene-Disease	CTD Gene-Disease	378	342	90.47%
Drugs-Disease and Chemical-Disease	CTD + CID: Chemical-Disease	3922	3485	88.86%

To determine if classification of overlaps can and made Table 8.

**Table 8:** Comparison of the text mining results of Drug-disease relation and existing databases (CID + CTD)

Relation in CID + CTD database	Relation in our results			
	Treatment	Cause	Other	Total
Treatment/therapeutic	1193	3	318	1514
Cause/induce	0	65	6	71
Other	82	28	2227	2337
Total	1275	96	2551	3922

By detailed analysis, we found our relation extraction method can accurately extract two entities in the same sentence, but there will be errors in the classification of the relations. The main reason is the inability to identify keywords in relation extraction.

At the same time, the other purpose of the construction of our knowledge graph is to extract knowledge that may be useful but not included in the existing data set in the vast ocean of data. For this, we compare the size of the data set with the existing biomedical common knowledge base and proposed new possible clinical medical research directions.

### StrokeKG

**Fig. 5:**
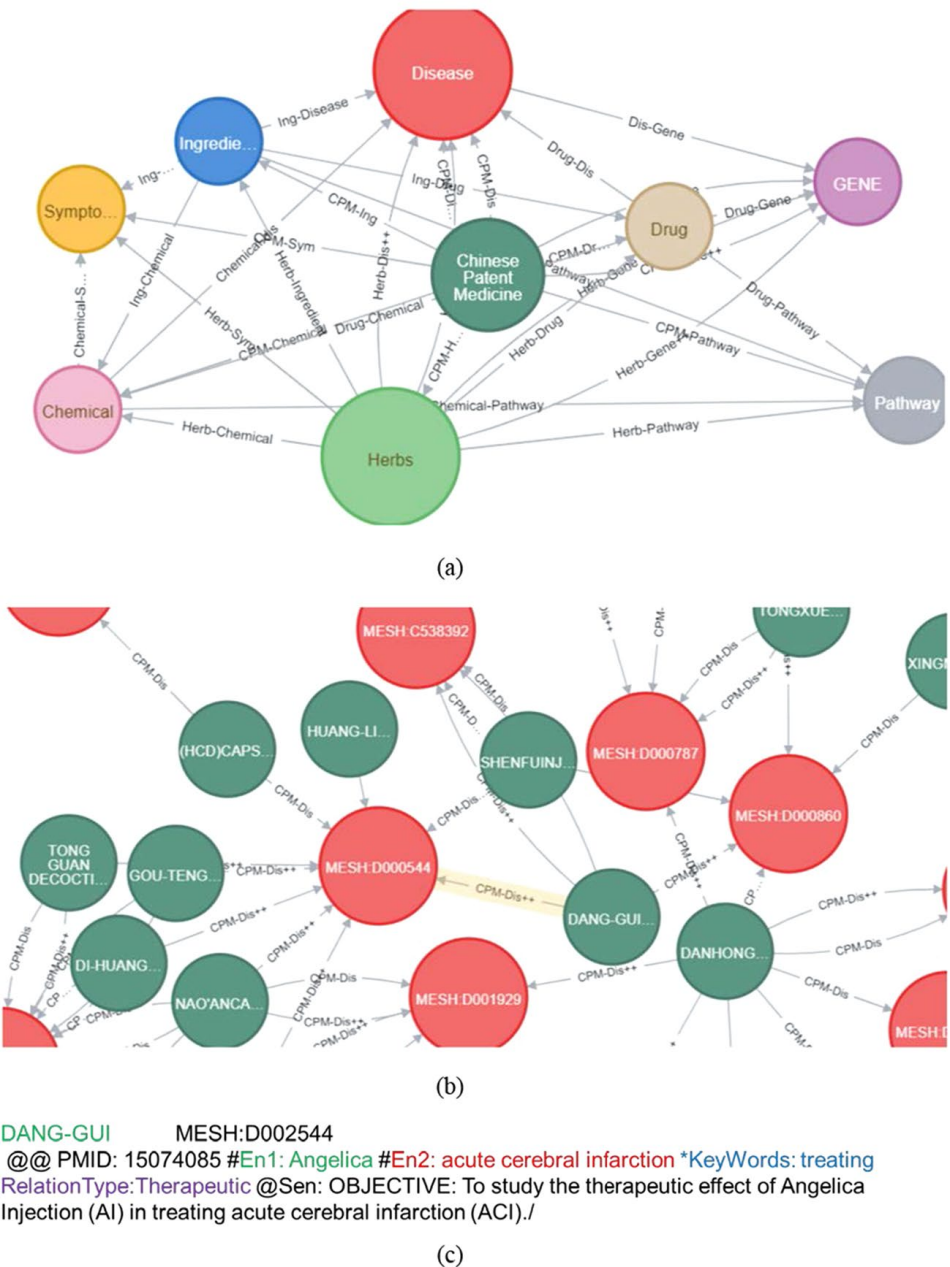
Screenshots of our knowledge graph. **a** Overall schema, **b** An example of details about the entity. **c** The relation between Danggui and acute cerebral infarction

StrokeKG (http://114.115.208.144:7474/browser/) contains a total of 46,983 entities belonging to K = 9 entity-types. The type-wise distribution of the entities. StrokeKG contains a total of 157,302 triplets belonging to R = 30 edge-types with 659,838 properties. A part of the results, as shown in Fig. 5a, using entities as graphs nodes, and the entities contain entity ID, entity name, and standard classification (MESH). As shown in Fig. 5b, the PMID number of the article where two entities co-exist is used as the edge of the graph. In particular, the edge also contains the keyword (RelationKeyword) extracted by PKDE4J and the relationship classification result (RelationType) based on the BERT model.

To enhance the effectiveness of our knowledge graph, we also annotated the reliable 32,031 nodes of 9 types and 4,800 relationships of 16 types with evidence from the entirely correct part of the evaluation results and the information in the existing database.

## Discussion

### Detail results of NER and RE

#### Stroke-related disease/symptoms

In total, we mined 4210 kinds of diseases (401,644 entity mentions) in downloaded documents. (Results detail shows in Additional file 1) According to our results, the expression of stroke in related literature includes synonyms(e.g., Apoplexy  (105), Brain ischemia (605), Cerebral ischemia (3183), Cerebrovascular Accident (227), Hemorrhage, Transient Ischemic Attack), abbreviations (e.g., CVA, TIA (722)), lexical changes, and word order changes. The generation of a stroke may be related to other diseases, such as atrial fibrillation (MESH:D001281,2732) diabetes (MESH:D006973, 2571), heart disease(MESH:D006333, 2590) etc., or it may have some sequelae after a stroke, such as acute gastrointestinal bleeding, hypertension, cerebral heart syndrome, pulmonary infection, and acute Pulmonary edema, etc. There are 728 types of symptoms (350,833 mentions), (Results detail shows in Additional file 2) Among them, aging (4103) mentioned in the 4041 abstract, which is also consistent with the fact that 64% of strokes occur in people aged 55 to 75 years. Virus (731) infection is a possible factor for sudden stroke. At the same time depression (1067), anxiety (301), and other unfavorable psychological conditions are also common complications that we need for stroke patients.

#### Stroke-related gene and relation between stroke-genes and stroke-related disease-genes

Gene mutations are related to the incidence of stroke. By relation extraction in disease-genes, we found 5953 types of genes (included 180,280 mentions). We linked 1238 diseases and 1574 genes, created 10,094 relationships. The results show that small changes in 588 genes can affect the risk of stroke and nearly 1000 genes affect stroke-related diseases. Specifically, changes in ACE (Angiotensin Converting Enzyme) (803), Collagen Binding Protein (437), or MTHFr (326) affect the risk of stroke. Secondly, VEGF (558) can be used as a drug target for the treatment of stroke patients. At the same time, the regulation of the brain protein of UCHL1, Hypoxia-inducible factor 1alpha (HIF-1α, 239) may be crucial for how nerve cells repair themselves after a stroke.

#### Western medicine for treating stroke and stroke-related disease

tmChem system successfully identified 11,129 types of Chemical entities (201,234 mentions) from the abstracts we downloaded. Among them, Ticlopidine, Nimodipine, Triphenyltetrazolium chloride has been mentioned many times and are ingredients contained in various medicines for the treatment of stroke and related diseases. It can be seen from the number of mentions that the aspirin (1475) is main chemical for relieving/ alleviating the risk of stroke, and angiotensin (435) causes vasoconstriction and increased blood pressure, which ultimately leads to stroke. Secondly, oxygen (2290), iron (1524) calcium (1918), glucose (1193) cholesterol (1177), nucleotide (1144), the index of these main compounds on the impact of human stroke and related diseases is the most concerned by the medical community.

According to the drug list provided by Drugbank, we have normalized and classified 2156 kinds of drugs for entities. In addition to the individual elements of statistics in the chemical, The drugs with the greatest impact on stroke are aspirin (DB00945,1475), warfarin (DB00682,1034), clopidogrel (DB00758,666).

#### TCM for treating stroke and stroke-related disease

We have identified 294 Chinese patent medicines that have played a role in the prevention and treatment of stroke and related diseases. From our mining results, GUALOUGUIZHI DECOCTION (10), KUDIEZIINJECTION(10), DANHONGINJECTION(20), and BUYANGHUANWU DECOCTION(36) are potent medicine in treating stroke. We also extracted 420 species of Herbs (11,671 mentions). DAN-SHEN (58), Chuan-Xiong (50), Dang-Gui (23), Huang-Lian (21), and Bai-Fu-Zi (19) are in various Chinese patent medicines or prescriptions for the treatment of stroke and related diseases. In ingredients extraction, except the ingredients like glucose (1947) cholesterol (1394) glutamate (767) dopamine (478), the unique ingredients in Chinese herbal medicine such as Hyperin (265) and catechol (207) are important for treating stoke-related diseases.

#### Pathways

In our results, a total of 105,337 pathways mentions were identified. In the subsequent relation extraction process, we use the results for analyzing what kind of molecular pair does the chemical in the medicine or the herbal ingredient play in the disease and identify what the key genes and pathways involved in stroke-related diseases are.

For example, the ERK1/2 activity generated by cytokines and free radicals or other inflammatory factors after stroke may worsen ischemic damage, whereas the ERK1/2 activity produced by exogenous growth factors, estrogen, and preconditioning favors neuroprotection.

#### Discover possible existing CPM to treat stroke

**Fig. 6:**
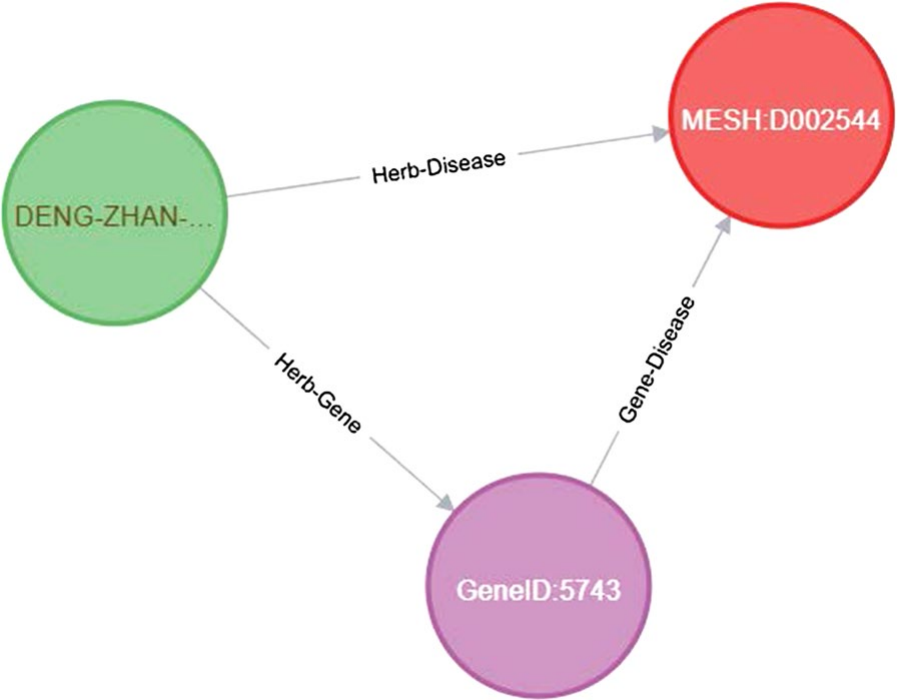
An example of expressed as a link between any pair of biological entities

StrokeKG Construction can discover possible existing drugs/CPM/herbs to treat stroke-related diseases to reduce the risk of stroke. Such a task can be expressed as a direct link prediction between the drug and disease entity, or indirectly expressed as a link between any pair of biological entities involved in a particular pathway. For example, *31348992 Intersection analysis between DZXXI's putative targets with ischemic stroke-associated genes identified two important targets (PTGS1, PTGS2)* (Fig. 6).

## Conclusions

In this study, we analyzed stroke-related literature with natural language processing, including named entity recognition and relation extraction. We showed that the-state-of-the-art text mining tools could efficiently extract the critical information hidden behind the unstructured data in the biomedical domain.

Through the knowledge base and knowledge graph, we have a clearer understanding of stroke-related diseases, symptoms, gene mutations that cause stroke, and the vital role of Chinese and Western medicine in preventing and treating stroke. We constructed StrokeKG, representing the relation among stroke-related entities successfully.

In future research, we will optimize the relationship mining model in the field of biomedicine, apply the model to all aspects of various diseases, and establish a larger and more comprehensive map of medical knowledge.

## Supplementary Information

**Additional file 1**. The list of stroke-related diseases.

**Additional file 2**. The list of stroke-related symptoms.

## Data Availability

The code files are available at: https://github.com/yangxi1016/Stroke/.

## Abbreviations

NER: Named Entity Recognition
RE: Relation Extraction
TCM: Traditional Chinese Medicine

## References

[1] Lau AY, Wong EH, Leung TW, Mok VC, Wong KS (2012). Intravenous alteplase for Chinese patients with stroke and borderline eligibility. J Clin Neurosci.

[2] Brainin M, Feigin VL, Norrving B, Martins SCO, Hankey GJ, Hachinski V (2020). Global prevention of stroke and dementia: the WSO declaration. Lancet Neurol.

[3] Peisker T, Koznar B, Stetkarova I, Widimsky P (2017). Acute stroke therapy: a review. Trends Cardiovasc Med.

[4] Ton G, Liao H-Y, Chiang J-H, Chen Y-H, Lee Y-C (2019). Chinese herbal medicine and acupuncture reduced the risk of stroke after Bell's Palsy: a population-based retrospective cohort study. J Altern Complement Med.

[5] Katan M, Luft A. Global burden of stroke. In: Seminars in neurology: 2018. Thieme Medical Publishers, 208–211. 10.1055/s-0038-1649503 29791947

[6] International Stroke Database [Internet]. 2006 Nov 1 [updated 2015 Jan 1; cited 2020 Dec 8]. Available from: http://www.nmr.mgh.harvard.edu/stroke/index.html

[7] OSR Acute Stroke Databases [Internet]. 2015 Oct 4 [updated 2021 May 1; cited 2021 May 6]. Available from: https://www.ices.on.ca/Research/Research-programs/Cardiovascular/Ontario-Stroke-Registry/OSR-Acute-Stroke-Databases

[8] SSNAP [Internet]. 2020 Jan 1 [updated 2021 May 4; cited 2021 May 6]. Available from: https://www.strokeaudit.org/

[9] Wang Q, Mao Z, Wang B, Guo L (2017). Knowledge graph embedding: a survey of approaches and applications. IEEE Trans Knowl Data Eng.

[10] Ji S, Pan S, Cambria E, Marttinen P, Yu PS. A survey on knowledge graphs: representation, acquisition and applications. arXiv preprint arXiv:2002.00388 2020. 10.1109/TNNLS.2021.3070843 33900922

[11] Wang X, He X, Cao Y, Liu M, Chua T-S. Kgat: knowledge graph attention network for recommendation. In: Proceedings of the 25th ACM SIGKDD international conference on knowledge discovery & data mining: 2019. 950–958.

[12] Bean DM, Wu H, Iqbal E, Dzahini O, Ibrahim ZM, Broadbent M, Stewart R, Dobson RJ (2017). Knowledge graph prediction of unknown adverse drug reactions and validation in electronic health records. Sci Rep.

[13] Gyrard A, Gaur M, Shekarpour S, Thirunarayan K, Sheth A. Personalized health knowledge graph. 2018. PMC8532078 34690624

[14] Ernst P, Siu A, Weikum G (2015). Knowlife: a versatile approach for constructing a large knowledge graph for biomedical sciences. BMC Bioinform.

[15] Mohamed SK, Nováček V, Nounu A (2020). Discovering protein drug targets using knowledge graph embeddings. Bioinformatics.

[16] Yuan J, Jin Z, Guo H, Jin H, Zhang X, Smith T, Luo J (2020). Constructing biomedical domain-specific knowledge graph with minimum supervision. Knowl Inf Syst.

[17] Teschke R, Zhang L, Long H, Schwarzenboeck A, Schmidt-Taenzer W, Genthner A, Wolff A, Frenzel C, Schulze J, Eickhoff A (2015). Traditional Chinese Medicine and herbal hepatotoxicity: a tabular compilation of reported cases. Ann Hepatol.

[18] Wu S, Wu B, Liu M, Chen Z, Wang W, Anderson CS, Sandercock P, Wang Y, Huang Y, Cui L (2019). Stroke in China: advances and challenges in epidemiology, prevention, and management. Lancet Neurol.

[19] Consortium GO (2015). Gene ontology consortium: going forward. Nucleic Acids Res.

[20] Schriml LM, Arze C, Nadendla S, Chang Y-WW, Mazaitis M, Felix V, Feng G, Kibbe WA. Disease ontology: a backbone for disease semantic integration. Nucleic Acids Res. 2012;40(D1):D940–6. 10.1093/nar/gkr972 PMC3245088 22080554

[21] Petry NM, Peirce JM, Stitzer ML, Blaine J, Roll JM, Cohen A, Obert J, Killeen T, Saladin ME, Cowell M (2005). Effect of prize-based incentives on outcomes in stimulant abusers in outpatient psychosocial treatment programs: a national drug abuse treatment clinical trials network study. Arch Gen Psychiatry.

[22] Gregory JK, Lachman N, Camp CL, Chen LP, Pawlina W (2009). Restructuring a basic science course for core competencies: an example from anatomy teaching. Med Teach.

[23] Yuanzhuo W, Yantao J, Zeya Z (2014). OpenKG-knowledge computing engine in the era of network big data. Commun Chin Comput Fed.

[24] Fauqueur J, Thillaisundara A, Togia T. Constructing large scale biomedical knowledge bases from scratch with rapid annotation of interpretable patterns. arXiv preprint arXiv:1907.01417 2019.

[25] Stebbing J, Phelan A, Griffin I, Tucker C, Oechsle O, Smith D, Richardson P. COVID-19: combining antiviral and anti-inflammatory treatments. Lancet Infect Dis. 2020. 10.1016/S1473-3099(20)30132-8 PMC7158903 32113509

[26] Sang S, Yang Z, Wang L, Liu X, Lin H, Wang J (2018). SemaTyP: a knowledge graph based literature mining method for drug discovery. BMC Bioinform.

[27] Cohen AM, Hersh WR (2005). A survey of current work in biomedical text mining. Brief Bioinform.

[28] Roux KJ, Kim DI, Burke B, May DG. BioID: a screen for protein–protein interactions. Curr Protoc Prot Sci. 2018;91(1):19.23.11–5. 10.1002/cpps.51 PMC6028010 29516480

[29] Lee K, Kim B, Choi Y, Kim S, Shin W, Lee S, Park S, Kim S, Tan AC, Kang J (2018). Deep learning of mutation-gene-drug relations from the literature. BMC Bioinform.

[30] Zhang Y, Lin H, Yang Z, Wang J, Zhang S, Sun Y, Yang L (2018). A hybrid model based on neural networks for biomedical relation extraction. J Biomed Inform.

[31] Yadav V, Bethard S. A survey on recent advances in named entity recognition from deep learning models. arXiv preprint arXiv:1910.11470 2019.

[32] Krauthammer M, Nenadic G. Term identification in the biomedical literature. J Biomed Inform. 37(6):512–26. 10.1016/j.jbi.2004.08.004 15542023

[33] Wei C-H, Kao H-Y, Lu Z. GNormPlus: an integrative approach for tagging genes, gene families, and protein domains. BioMed Res Int. 2015;2015. 10.1155/2015/918710 PMC4561873 26380306

[34] Leaman R, Islamaj Doğan R, Lu Z (2013). DNorm: disease name normalization with pairwise learning to rank. Bioinformatics.

[35] Dang TH, Le H-Q, Nguyen TM, Vu ST (2018). D3NER: biomedical named entity recognition using CRF-biLSTM improved with fine-tuned embeddings of various linguistic information. Bioinformatics.

[36] Gerner M, Nenadic G, Bergman CM (2010). LINNAEUS: a species name identification system for biomedical literature. BMC Bioinform.

[37] Perera D, Poulos RC, Shah A, Beck D, Pimanda JE, Wong JW (2016). Differential DNA repair underlies mutation hotspots at active promoters in cancer genomes. Nature.

[38] Leaman R, Wei C-H, Lu Z (2015). tmChem: a high performance approach for chemical named entity recognition and normalization. J Cheminform.

[39] Rocktäschel T, Weidlich M, Leser U (2012). ChemSpot: a hybrid system for chemical named entity recognition. Bioinformatics.

[40] Wu C, Schwartz J-M, Brabant G, Peng S-L, Nenadic G (2015). Constructing a molecular interaction network for thyroid cancer via large-scale text mining of gene and pathway events. BMC Syst Biol.

[41] Wang W, Yang X, Yang C, Guo X, Zhang X, Wu C (2017). Dependency-based long short term memory network for drug–drug interaction extraction. BMC Bioinform.

[42] Van Mulligen EM, Fourrier-Reglat A, Gurwitz D, Molokhia M, Nieto A, Trifiro G, Kors JA, Furlong LI (2012). The EU-ADR corpus: annotated drugs, diseases, targets, and their relationships. J Biomed Inform.

[43] Trifirò G, Patadia V, Schuemie MJ, Coloma PM, Gini R, Herings R, Hippisley-Cox J, Mazzaglia G, Giaquinto C, Scotti L. EU-ADR healthcare database network vs. spontaneous reporting system database: preliminary comparison of signal detection. Stud Health Technol Inform. 2011;166:25–30. 21685607

[44] Junge A, Jensen LJ (2020). CoCoScore: context-aware co-occurrence scoring for text mining applications using distant supervision. Bioinformatics.

[45] Sarhan I, El-Sonbaty Y, El-Nasr MA. Semi-supervised pattern based algorithm for arabic relation extraction. In: 2016 IEEE 28th international conference on tools with artificial intelligence (ICTAI): 2016. IEEE: 177–183.

[46] Song M, Kim WC, Lee D, Heo GE, Kang KY (2015). PKDE4J: Entity and relation extraction for public knowledge discovery. J Biomed Inform.

[47] Ravikumar K, Rastegar-Mojarad M, Liu H. BELMiner: adapting a rule-based relation extraction system to extract biological expression language statements from bio-medical literature evidence sentences. Database. 2017;2017. 10.1093/database/baw156 PMC5467463 28365720

[48] Kim J-D, Ohta T, Pyysalo S, Kano Y. 2009. Overview of bionlp’09 shared task on event extraction. In: Proceedings of natural language processing in biomedicine (BioNLP) NAACL 2009 workshop. Citeseer.

[49] Liu C, Sun W, Chao W, Che W. Convolution neural network for relation extraction. In: International conference on advanced data mining and applications: 2013. Springer: 231–242.

[50] Zhang D, Wang D: Relation classification via recurrent neural network. arXiv preprint arXiv:1508.01006 2015.

[51] Xi E, Bing S, Jin Y: Capsule network performance on complex data. arXiv preprint arXiv:1712.03480 2017.

[52] Sun M, Zhao S, Gilvary C, Elemento O, Zhou J, Wang F. Graph convolutional networks for computational drug development and discovery. Brief Inform. 2019. 10.1093/bib/bbz042 31155636

[53] Zitnik M, Agrawal M, Leskovec J (2018). Modeling polypharmacy side effects with graph convolutional networks. Bioinformatics.

[54] Yu X, Hu W, Lu S, Sun X, Yuan Z. BioBERT Based Named Entity Recognition in Electronic Medical Record. In: 2019 10th international conference on information technology in medicine and education (ITME): 2019. IEEE: 49–52.

[55] Huang L, Xie D, Yu Y, Liu H, Shi Y, Shi T, Wen C. TCMID 2.0: a comprehensive resource for TCM. Nucleic Acids Res. 2017;46(D1):D1117–20. 10.1093/nar/gkx1028 PMC5753259 29106634

[56] Zhang R-z, Yu S-j (2017). Bai H, Ning K: TCM-Mesh: the database and analytical system for network pharmacology analysis for TCM preparations. Sci Rep.

[57] Xu H-Y, Zhang Y-Q, Liu Z-M, Chen T, Lv C-Y, Tang S-H, Zhang X-B, Zhang W, Li Z-Y, Zhou R-R (2019). ETCM: an encyclopaedia of traditional Chinese medicine. Nucleic Acids Res.

[58] Ru J, Li P, Wang J, Zhou W, Li B, Huang C, Li P, Guo Z, Tao W, Yang Y (2014). TCMSP: a database of systems pharmacology for drug discovery from herbal medicines. J Cheminform.

[59] Taboureau O, Nielsen SK, Audouze K, Weinhold N, Edsgärd D, Roque FS, Kouskoumvekaki I, Bora A, Curpan R, Jensen TS. ChemProt: a disease chemical biology database. Nucleic Acids Res. 2010;39(suppl_1):D367–72. 10.1093/nar/gkq906 PMC3013776 20935044

[60] Mattingly C, Rosenstein M, Colby G, Forrest J, Boyer J (2006). The Comparative Toxicogenomics Database (CTD): a resource for comparative toxicological studies. J Exp Zool Part A Comp Exp Biol.

[61] Herrero-Zazo M, Segura-Bedmar I, Martínez P, Declerck T (2013). The DDI corpus: An annotated corpus with pharmacological substances and drug–drug interactions. J Biomed Inform.

[62] Kim B, Choi W, Lee H. A corpus of plant–disease relations in the biomedical domain. PLoS ONE 2019;14(8). 10.1371/journal.pone.0221582 PMC6713337 31461491

[63] Wei C-H, Kao H-Y, Lu Z (2013). PubTator: a web-based text mining tool for assisting biocuration. Nucleic Acids Res.

[64] Loper E, Bird S. NLTK: the natural language toolkit. arXiv preprint arXiv:cs/0205028 2002.

[65] Luo L, Yang Z, Yang P, Zhang Y, Wang L, Lin H, Wang J (2018). An attention-based BiLSTM-CRF approach to document-level chemical named entity recognition. Bioinformatics.

[66] Li J, Sun Y, Johnson RJ, Sciaky D, Wei C-H, Leaman R, Davis AP, Mattingly CJ, Wiegers TC, Lu Z. BioCreative V CDR task corpus: a resource for chemical disease relation extraction. Database. 2016;2016. 10.1093/database/baw068 PMC4860626 27161011

