# Correction to: Mining a stroke knowledge graph from literature

**DOI:** 10.1186/s12859-021-04502-z

**Published:** 2021-12-08

**Authors:** Xi Yang, Chengkun Wu, Goran Nenadic, Wei Wang, Kai Lu

**Affiliations:** 1 College of Computer, National University of Defence Technology, Changsha, 410073 China; 2 State Key Laboratory of High-Performance Computing, National University of Defence Technology, Changsha, 410073 China; 3 Department of Computer Science, University of Manchester, Manchester, M13 9PL UK

## Correction to: BMC Bioinformatics (2021) 22:387 10.1186/s12859-021-04292-4

Following publication of the supplement article [1], the authors identified an error in the equal contributors.

After a sincere and thorough discussion among the authors it was decided that Chengkun Wu should not be listed as the 1st co-author. All authors agreed that Xi Yang should be the only 1st author of the paper.

The author group has been updated above and the original article [1] has been corrected.
